# Ionic liquid-stabilized non-spherical gold nanofluids synthesized using a one-step method

**DOI:** 10.1186/1556-276X-7-583

**Published:** 2012-10-23

**Authors:** Hao Zhang, Hua Cui, Shiwei Yao, Kelong Zhang, Haikun Tao, Haibo Meng

**Affiliations:** 1 Laboratory on Steam Power System, Wuhan Second Ship Design and Research Institute, Wuhan, Hubei, 430064, People’s Republic of China; 2 CAS Key Laboratory of Soft Matter Chemistry, Department of Chemistry, University of Science and Technology of China, Hefei, Anhui, 230026, People's Republic of China

**Keywords:** Ionic liquid, Gold, Nanofluid, One-step method, Non-spherical

## Abstract

Ionic liquid (IL)-stabilized non-spherical gold nanofluids have been synthesized by a one-step method in aqueous solution. The whole reaction proceeded in room temperature. In the presence of amino-functionalized ionic liquids, gold nanofluids with long-wave surface plasmon resonance (SPR) absorption (>600 nm) could be obtained by adopting tannic acid as the reductant. The specific SPR absorption was related to the non-spherical gold nanoparticles including gold triangle, decahedra, and icosahedra nanocrystals. All the nanocrystals were observed by transmission electron microscopy. It was deduced that the formation of non-spherical gold nanofluids was related to the hydroxyls in tannic acid while IL acted as the synthesis template.

## Background

Heat exchange efficiency is an important property of working media used in heat transfer devices. It affects the economic attractiveness and general performance of the related devices. In the past several years, great efforts were made to improve the efficiency of the working media while nanofluids were explored. In the work of Choi et.al [1] and Eastman et.al [2], nanofluids showed great enhancements in thermal conductivity when small amounts of metallic or other nanoparticles were dispersed in common heat transfer fluids. When the composition of the nanofluids was settled, the heat transfer efficiency greatly depended on the synthesis procedure.

The current synthesis methods can be generally divided into two types, namely, the two-step method and the one-step method [3,4]. For the two-step method, nanoparticles are either synthesized or purchased first in the form of dry powders, and the nanofluid formulation process involves the proper separation of the aggregated dried particles into individual particles and keeping them from re-agglomeration under suitable ionic or surfactant conditions [5]. It is a very complicated process, and the performance of the obtained nanofluid will be affected by several factors. In contrast, the one-step method requires less operation. Nanofluids are synthesized through physical or chemical reactions [5,6]. The advantage of this process lies in the minimized nanoparticle agglomeration. The stability of nanofluids is guaranteed by proper surface functionalization without involving mechanical facilities. Since noble metal shows chemical and physical affinity with many species, the one-step method is usually adopted to fabricate noble metal nanofluids such as gold nanofluids (AuNFs). However, this approach suffers the problem of impurities. For example, residual reactants are generally left in the nanofluids because of incomplete reaction or stabilization. It is difficult to elucidate the nanoparticle effect without eliminating this impurity effect [5].

Ionic liquid (IL) has been widely studied due to its unique physicochemical properties such as negligible vapor pressure, non-flammability, high ionic conductivity, low toxicity, good solvent for organic and inorganic molecules, high thermal stability, and wide electrochemical window [7-9]. IL containing imidazole ring shows great applicable potential and can be used as the stabilizer of metal nanoparticles [10,11]. The as-prepared nanofluids can be easily dispersed in aqueous solution, which implies a significant applicable value in thermodynamics. Imidazole IL can also be functionalized with different groups such as carboxyl and amido. Since both carboxyl and amido have a special affinity with noble metal, the functionalized IL-stabilized metal nanofluids have an improved stability [12-14]. For example, imidazole IL-stabilized AuNFs were synthesized in a one-phase and/or two-phase method and had a remarkable thermal conductivity [15-17].

Fundamental research and practical applications of AuNFs are now becoming attractive subjects due to their promising chemical, biological, and physical properties [18,19]. Frens [20] firstly offered a simple and effective method to synthesize spherical gold nanoparticles. Because the properties of gold nanomaterials were greatly influenced by their size and morphology, the synthesis of AuNFs of controllable shape was a general routine to prepare gold nanomaterials with valuable properties [21]. Non-spherical AuNFs such as nanorods [22], nanoslices [23], nanoprisms [24], nanoflowers [25], and nanopolyhedrons [26] could be obtained in different ways [27,28]. However, the reported synthesis processes were very complicated. One or more special conditions such as temperature, pressure, organic solvent, and assisted technique were required.

In our previous work, we synthesized spherical AuNFs in a simple way [29]. NaBH_4_ or trisodiumcitrate was used as the reductant. In the presence of IL, spherical gold nanoparticles were dispersed in aqueous solution. The average diameter of the spheres was from 3.5 to 100 nm. The surface plasmon resonance (SPR) absorption peaks of the prepared colloids were observed between 512 and 553 nm, which belonged to the typical absorption of spherical good nanoparticles with different sizes [30,31]. Compared with trisodiumcitrate-stabilized gold nanoparticles, IL-stabilized AuNFs exhibited some advantages such as decrease in aggregation and increase in stability [29]. Moreover, IL belonged to inert ionic compounds, which meant that it would be stable in aqueous solution without decomposition or phase transformation.

In this manuscript, non-spherical AuNFs are synthesized in common conditions. AuNFs consisted of various nanocrystals with different compositions and size can be synthesized by simply changing the amount of reductant during a one-step reacting process. IL is used as the stabilizer, which can improve the stability of AuNFs in aqueous solution. The non-special conditions such as room temperature, atmospheric pressure, and aqueous solution are available everywhere, which would make the nanofluids convenient for further usage.

## Methods

### Chemicals and solutions

**Figure 1:**
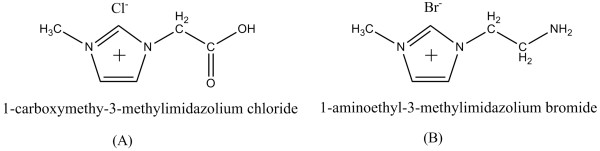
**Molecular structures of the used ILs.** (**A**) 1-carboxymethy-3-methylimidazolium chloride and (**B**) 1-aminoethyl-3-methylimidazolium bromide.

A 1% (*w*/*w*) trisodiumcitrate solution was prepared by dissolving trisodiumcitrate solids (Shanghai Reagent, Shanghai, China) in purified H_2_O. A 1% (*w*/*w*) tannic acid (TA) solution was prepared by dissolving TA solids (Shanghai Reagent, Shanghai, China) in purified H_2_O. A HAuCl_4_ stock solution (2 ‰ HAuCl_4_, *w*/*w*) was prepared by dissolving 1.0 g of HAuCl_4_·4H_2_O (Shanghai Reagent, Shanghai, China) in 412 mL of purified water and was stored at 4°C. A 1-carboxymethy-3-methylimidazolium chloride ([CMMIM]·Cl, Figure 1A) stock solution (1.0 mmol·L^−1^) was prepared by dissolving 0.0875 g of [CMMIM]·Cl (Shanghai Shyfhx Reagent, Shanghai, China) in 500 mL of purified water. A 1-aminoethyl-3-methylimidazolium bromide ([AEMIM]·Br, Figure 1B) stock solution (2.0 mmol·L^−1^) was prepared by dissolving 0.01 g of [AEMIM]·Br (Shanghai Shyfhx Reagent, Shanghai, China) in 25 mL of purified water. Polyvinylpyrrolidone (PVP) and NaBr were obtained from Shanghai Reagent (Shanghai, China). All reagents were of analytical grade and used as received without further purification. Ultrapure water was prepared through a Millipore (Billerica, MA, USA) Direct-Q 3 system and used throughout.

### Synthesis of AuNFs

All glassware used in the following procedures was cleaned in a bath of freshly prepared HNO_3_/HCl (1:3, *v*/*v*), rinsed thoroughly in purified water, and dried prior to use. A mixture of different volumes of TA solution (1%, *w*/*w*) and different volumes of stabilization reagent was added rapidly into the HAuCl_4_ solution (0.01%, *w*/*w*) with vigorous stirring. The total volume of the mixture should be settled as 50 mL. The mixture was stirred continuously during which time a color change from yellow to red was observed. When the color did not change anymore, AuNFs were well prepared and the dispersions were stored at 4°C for future use. Commonly, the whole reaction cost about 0.5 h.

### Characterization of AuNFs

As-synthesized AuNFs were subsequently characterized by high-resolution transmission electron microscopy (HRTEM) (JEOL-2010, Jeol Ltd., Akishima, Tokyo, Japan) and UV-visible (vis) spectroscopy (8453 UV–vis spectrophotometer, Agilent Technologies, Inc., Santa Clara, CA, USA). When a nanocolloid sample was characterized by HRTEM, the liquid sample was dipped onto a copper grid. After the solvent of the sample evaporates from the grid, colloids remained and could be easily observed by microscopy. The obtained images showed a partial area of the copper grid. The HRTEM photomicrographs were further edited by Adobe Photoshop CS (Adobe Systems Inc., San Jose, CA, USA).

## Results and discussion

### Typical functionalized IL-stabilized AuNFs

**Figure 2:**
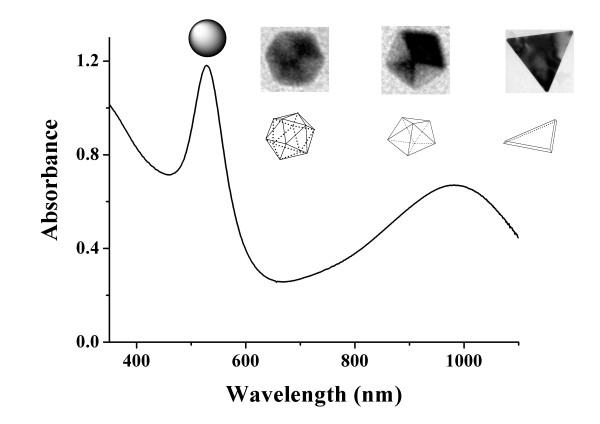
**UV–vis-NIR absorbance spectroscopy of synthesized AuNFs.** Experimental conditions 0.20 mL 1% (*w*/*v*) TA, 0.50 mL 2.0 mmol·L^−1^ [AEMIM]·Br.

Typical [AEMIM]·Br-stabilized AuNFs were synthesized while TA was used as reductant. Figure 2 shows the SPR absorption graph of the AuNFs. The sharp peak around 528 nm was attributed to spherical gold nanoparticles [30,31]. Different from the IL-stabilized AuNFs deoxidized by trisodiumcitrate or NaBH_4_, a notable flat peak could be observed in the long-wave area. The maximum absorbance was around 984 nm. It was reported that the long-wave absorption of the AuNF SPR spectroscopy was attributed to non-spherical nanoparticles such as nanoflowers [31], triangles [32,33], decahedrons [26,32], and so on. In order to find out the source of the absorption peak, HRTEM photos were taken.

**Figure 3:**
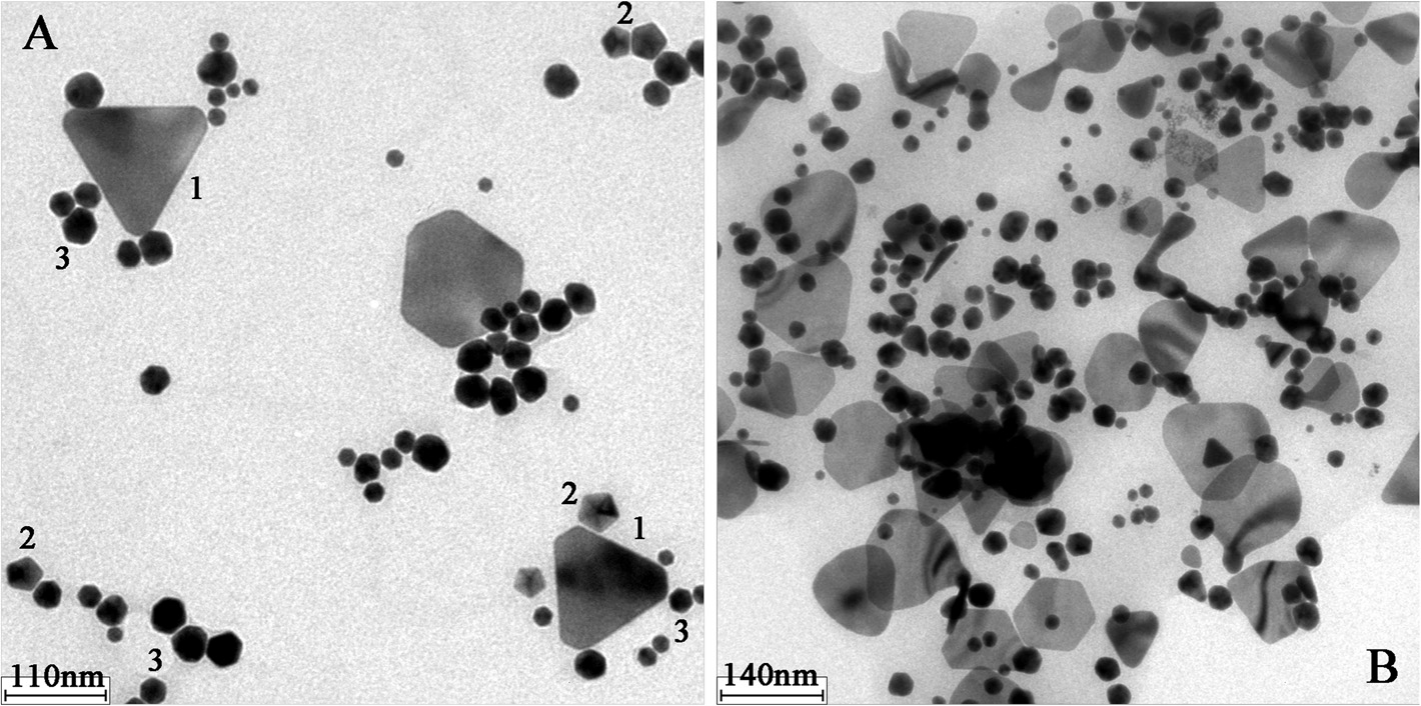
**TEM images of one AuNF sample.** Experimental conditions 0.20 mL 1% (*w*/*v*) TA, 0.50 mL 2.0 mmol·L^−1^ [AEMIM]·Br.

Figure 3 shows the HRTEM images of as-prepared AuNFs. In Figure 3, the two images were taken from different locations of one AuNF sample. From the pictures, three kinds of non-spherical particles could be found in Figure 3A,B. The triangle particle was marked with ‘1’, and the decahedron was marked with ‘2’ in Figure 3A. A single icosahedron particle had many edges and corners, which made it a complicated solid morphology and was similar to a sphere in photos. The icosahedron was illustrated in Figure 2 and was marked with ‘3’ in Figure 3A. In general, the HRTEM images only represent a partial area microcosmically. Different regions might have different images. The SPR absorption is a property of metal nanoparticles triggered by the small size effect. It represents an optical phenomenon which is contributed by each of the particles. Therefore, it demonstrates the macroscopical status of the AuNFs. With the combination of Figures 2 and 3, it could be concluded that the peak around 528 nm was due to the SPR absorption of spherical particles, while the peak around 984 nm was due to the SPR absorption of non-spherical particles including triangles, decahedrons, and icosahedrons. Species of different morphology and abundance were mixed very well with each other.

### Effect of different stabilizers

As described, the AuNFs were synthesized by a relatively rapid and simple reaction. In order to investigate the formation mechanism of multiple morphologies, reagents involved in the synthesis process were changed slightly. The SPR absorption was used to characterize the AuNFs macroscopically. The long-wave absorbance indicated the relative abundance of the non-spherical particles.

**Figure 4:**
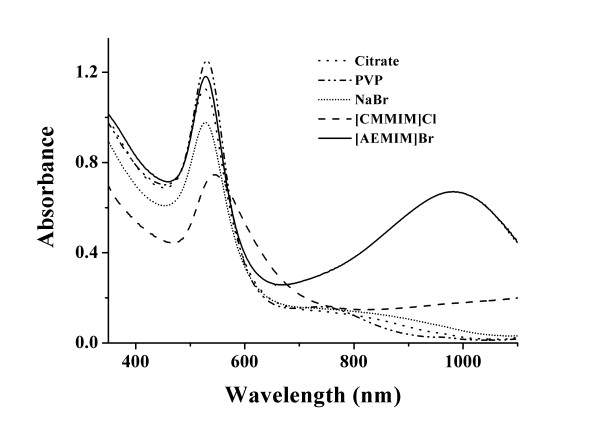
**Effects of different stabilizers on UV–vis-NIR absorbance spectroscopy of AuNFs.** Experimental conditions, 0.20 mL 1% (*w*/*v*) TA as reducing agent. Solid line, 0.50 mL 2.0 mmol·L^−1^ [AEMIM]·Br; dash line, 1.0 mL 2.0 mmol·L^−1^ [CMMIM]·Cl; short dot line, 0.50 mL 1.0 mmol·L^−1^ NaBr; dash dot line, 1.5 mg PVP; and dot line, 0.25 mL 1% (*w*/*v*) trisodiumcitrate.

Figure 4 shows the effects of different stabilizers on the morphology of AuNFs. Except for the obvious peak of solid line, no long-wave peak (from 600 to 1,100 nm) could be found while other reagents were used as stabilizer. When a carboxylic IL was the stabilizer, absorption could be found from 800 to 1,100 nm, which was shown by a dash line in Figure 4. The absorbance was much weaker than that of the [AEMIM]·Br-stabilized AuNFs, which implied a less abundance of non-spherical particles. In order to exclude the influence of bromine anion, NaBr was also added into the mixture. It was found that no absorbance in the long-wave area was observed, as shown in Figure 4.

In conclusion, no non-spherical particles were formed when the synthesis process underwent without IL. When IL was used as the stabilizer, long-wave absorbance appeared. The absorbance was not related to bromine anion. The imidazole cation played an important role in the formation of non-spherical particles, and the amino IL displayed a better performance than the carboxyl IL. It was reported [29,34] that the amino group could form the covalent bond (Au-N) with gold atom. The carboxyl functional group also had a conjugated interaction with gold atom [35,36]. The covalent bond was a more powerful interaction. Therefore, [AEMIM]·Br could be stabilized in an efficient way and has made the non-spherical particles grow much easier.

### Effect of the concentration of amino ILs

**Figure 5:**
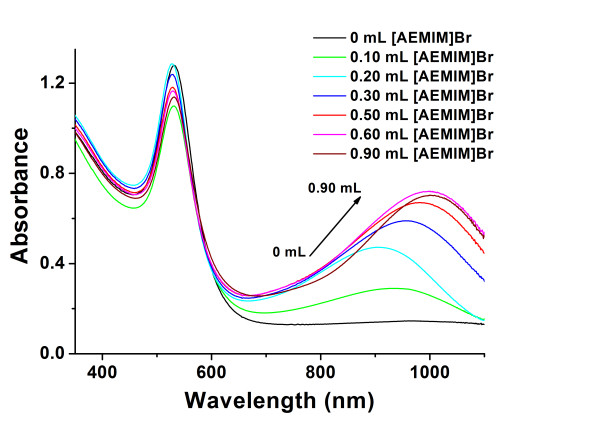
**Effects of the amount of IL on UV–vis-NIR absorbance spectroscopy of AuNFs.** Experimental condition: different volumes of 2.0 mmol·L^−1^ [AEMIM]·Br as the capper and 0.20 mL 1% (*w*/*w*) TA as the reducing agent.

Herein, the effect of the concentration of amino ILs was examined and shown in Figure 5. With the fixed amount of TA, different AuNFs were obtained by changing the volume of the [AEMIM]·Br stock solution. No obvious absorption existed in the absence of [AEMIM]·Br. A few volumes of [AEMIM]·Br would induce the absorbance peak around 900 nm. The peak value increased with the volume of stabilizer. When the volume was settled at 0.5 mL or more, the peak value and position changed slightly. The experimental results testified the inducement effect of amino ILs.

### Effect of the concentration of reductant

**Figure 6:**
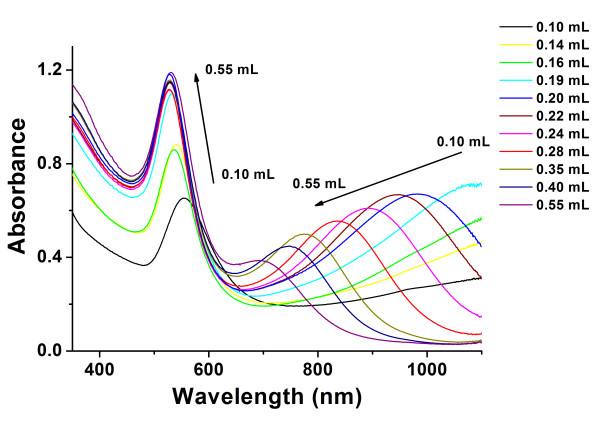
**Effects of the amount of reducing agent on UV–vis-NIR absorbance spectroscopy of AuNFs.** Experimental conditions different volume of 1% (*w*/*v*) TA as the reducing agent and 0.50 mL 2.0 mmol·L^−1^ [AEMIM]·Br as the capper.

In ‘Effect of the concentration of amino ILs’ section, the concentration of stabilizer was changed and examined in sequence. Amino IL could induce long-wave absorption. However, the peak position mostly decided by the morphology of AuNFs could not be influenced remarkably by the changing amount of stabilizer. For the usage of nanofluids, various morphologies might increase the heat transfer efficiency [37]. Therefore, it appeared valuable to synthesize nanofluids with different absorption values. The amount of TA was changed orderly with the fixed volume of amino IL in the following experiment. Different absorption curves were obtained as shown in Figure 6. It should be mentioned that the synthesis process required a longer time if the amount of TA decreased. Meanwhile, the total absorbance of AuNFs in the UV–vis-near infrared (NIR) area decreased. When the volume of TA was less than 0.19 mL, the long-wave absorption peak could not be observed. A peak appeared at 1,080 nm when 0.19 mL of TA stock solution was added. With the increase of TA, the peak position kept moving toward the short-wave area. The peaks were observed at 984 nm (0.20 mL TA), 946 nm (0.22 mL TA), 893 nm (0.24 mL TA), 838 nm (0.28 mL TA), 775 nm (0.35 mL TA), 747 nm (0.40 mL TA), and 697 nm (0.55mL TA) separately. Among all the curves in Figure 6, the peak at 528 nm always existed. The peak was induced by the spherical gold nanoparticles [29,31], which meant that the spheres were always formed in different chemical conditions.

**Figure 7:**
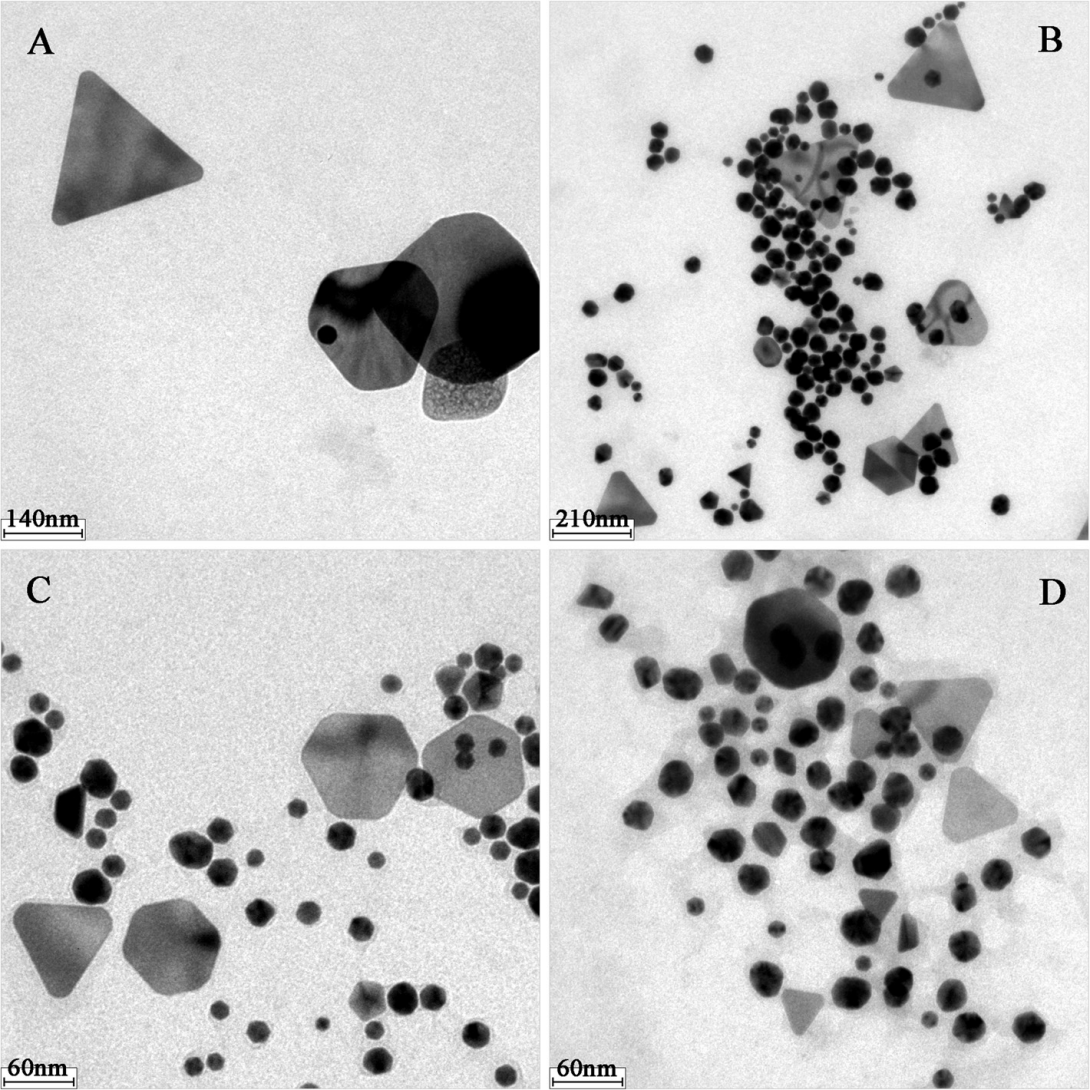
**TEM images of synthesized AuNFs.** Experimental conditions 0.50 mL 2.0 mmol·L^−1^ [AEMIM]·Br as the capper and different volumes of 1% (*w*/*v*) TA as reducing agent: (**A**) 0.10 mL TA, (**B**) 0.15 mL TA, (**C**) 0.24 mL TA, (**D**) 0.28 mL TA.

The difference in the SPR spectrogram indicated the difference in morphology. Therefore, it was necessary to find out the evidence from the transmission electron microscopy (TEM) photos. Figure 7 shows the partial images of four different samples. It was found that the sol of AuNFs reduced by 0.10 mL TA was very rare. As shown in Figure 7A, a few triangles scattered on the underlay here and there. When the reductant increased, more sol particles could be found gathering together, as shown in Figure 7B. The size of the triangle decreased with the increase of TA attending the reaction. Besides, several polygonal pieces and irregular polyhedrons existed in Figure 7. It is known that both thermodynamic balance and complicated chemical conditions would greatly affect the growing kinetics of nanocrystal. The final influence would appear as different growing velocity in various crystal lattice directions. Therefore, the obtained crystals might have defects somewhere, and anisotropic triangles or polyhedrons were formed.

It is clear that the SPR property of gold nanomaterial is related to its morphology and size of particles. For the regular gold nanomaterial, the general relationship between SPR property and geometric parameter was established theoretically [38] and experimentally [39]. Millstone and co-workers [24] pointed out that the gold nanotriangles had SPR absorbance in the NIR area. Based on the side length of the triangle, the peak would be distributed from 800 nm (side length 60 nm) to 1,300 nm (side length 150 nm). Shankar and co-workers [33] also reported that the SPR absorbance of gold nanotriangles started from 650 nm and increased with the wavelength. A maximum appeared near 1,300 nm. The absorbance in the long-wave area was very similar with the curves in Figure 6. For the other crystals in AuNFs, there were also some research about its SPR property. It was reported [26] that the SPR absorption of gold nanodecahedrons was greatly concerned with its size. The absorbance appeared from 700 to 800 nm, which was confirmed by the calculating results of discrete dipole approximation [26]. The icosahedrons had a complicated tridimensional structure. Various factors would affect the growth of a perfect icosahedron crystal, which made the icosahedrons grow into a sphere easily. Therefore, its SPR absorption appeared very similar to that of the spherical gold nanoparticles [26].

According to the references and TEM images in Figure 7, it could be deduced that the long-wave absorbance peak might exist in the area from 1,100 to 1,300 nm when the volume of TA was less than 0.19 mL. Our spectrophotometer could not collect the data from 1,100 to 1,300 nm. When the peak shifted to 1,080 nm (0.19 mL TA was added), the maximum datum was collected very well as shown in Figure 6. The peak continued shifting with the amount of TA increased. The observed shifts were from 1,080 to 697 nm. Meanwhile, gold nanotriangles and nanodecahedrons were clearly identified in TEM images. With the cited literatures, we concluded that the shifts corresponded to the change of size and abundance of gold nanotriangles and nanodecahedrons. In other words, AuNFs consisted of various nanocrystals with different compositions, and size could be synthesized by simply changing the amount of reductant during the one-step reacting process.

### A testified experiment on the effect of amino IL

**Figure 8:**
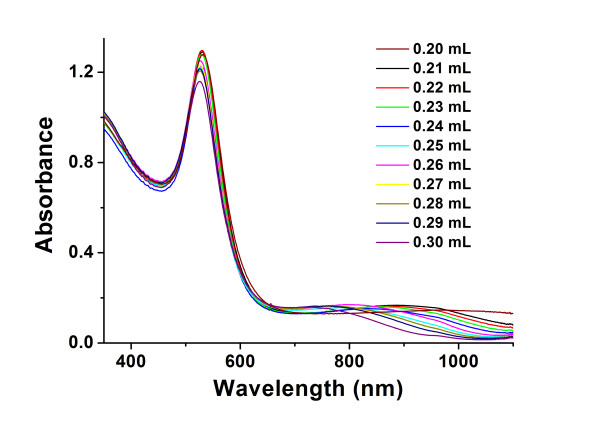
**UV–vis-NIR absorbance spectroscopy of the AuNFs synthesized without ionic liquid.** Experimental conditions different volumes of 1% (*w*/*v*) TA as reducing agent.

In the previous sections, we deduced that the amount of TA affected the formation of non-spherical AuNFs. A testified experiment was designed to exclude the effect of amino IL; it is shown in Figure 8. When [AEMIM]·Br was absent, no obvious long-wave peak could be found. The change of TA only affected the absorbance slightly and could not demonstrate anything significant.

### The formation of non-spherical AuNFs

**Figure 9:**
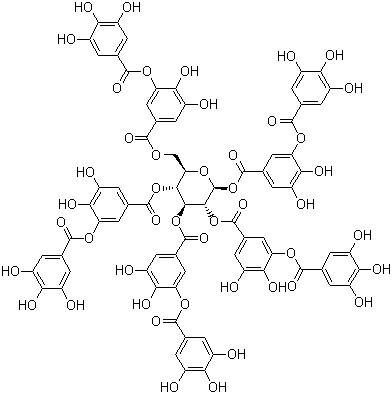
Structure of tannic acid.

In our previous work [29], spherical AuNFs were obtained using NaBH_4_ or trisodiumcitrate as reductant in the presence of IL. Therefore, the formation of non-spherical nanoparticles was definitely concerned with the reductant TA. It has been reported [32,40] that different kinds of non-spherical gold nanoparticles could be synthesized when the reductant had several hydroxys. Figure 9 shows the reductant used in this manuscript. The hydroxys in TA made the formation of special crystals possible.

IL could stabilize gold nanoparticles by static interaction [41]. The imidazole ring was a positive ion and had a special static property. It preferred to interact with a metal atom from a special angle [42,43]. Besides, IL molecules could form a specific structure by π-π stack interaction. The specific structure acted as a perfect mode for the synthesis of non-spherical AuNFs [44-46]. Therefore, we deduced that the special angle and structure caused by IL would result in the asymmetry of electric field around gold nanoparticles. The properties decreased the growing energy of some special crystal angle and prevented the nanoparticles from aggregating together. Non-spherical AuNFs were formed subsequently.

## Conclusions

In this manuscript, a one-step method in the synthesis of AuNFs was well established. Non-spherical AuNFs were obtained in aqueous solution. The results were observed by the usage of HRTEM and UV–vis spectroscopy. According to the experiment, some conclusions could be reached and are shown as follows:

1. Both TA and amino ILs were necessary in the formation of non-spherical AuNFs.

2. The amount of amino ILs could increase the abundance of non-spherical nanoparticles. However, a limitation existed. When the amount of amino ILs reached a fixed value, the abundance would no longer increase.

3. Amino ILs had a better performance than carboxyl ILs in the synthesis process of non-spherical AuNFs.

4. The alteration of TA volume would change the composition and size of non-spherical nanoparticles.

The results enrich the research of surface functionalization of gold nanoparticles. Different shapes of IL-stabilized AuNFs can easily be prepared in room temperature. Moreover, the as-prepared AuNFs are dispersed in aqueous solution, which makes it more valuable in industrial applications. Besides the heat transfer efficiency, more research should also be focused in the stability of AuNFs in the near future.

## Competing interests

The authors declare that they have no competing interests.

## Authors’ contributions

HZ performed the experiments and helped draft the manuscript. HC proposed the idea and designed the experiments. SY and KZ collected information and finalized the manuscript. HT and HM helped in making the experiments. All authors read and approved the final manuscript.
